# Health Care Reform: Understanding Individuals' Attitudes and Information Sources

**DOI:** 10.1155/2014/813851

**Published:** 2014-06-19

**Authors:** Carolyn K. Shue, Kerry Anne McGeary, Ian Reid, Jagdish Khubchandani, Maoyong Fan

**Affiliations:** ^1^Department of Communication Studies, Ball State University, Muncie, IN 47306, USA; ^2^Department of Economics, Ball State University, Muncie, IN 47306, USA; ^3^Global Health Institute, Ball State University, Muncie, IN 47306, USA; ^4^Department of Physiology and Health Science, Ball State University, Muncie, IN 47306, USA

## Abstract

Since passage of the Affordable Care Act (ACA) was signed into law by President Barrack Obama, little is known about state-level perceptions of residents on the ACA. Perceptions about the act could potentially affect implementation of the law to the fullest extent. This 3-year survey study explored attitudes about the ACA, the types of information sources that individuals rely on when creating those attitudes, and the predictors of these attitudes among state of Indiana residents. The respondents were split between favorable and unfavorable views of the ACA, yet the majority of respondents strongly supported individual components of the act. National TV news, websites, family members, and individuals' own reading of the ACA legislation were identified as the most influential information sources. After controlling for potential confounders, the respondent's political affiliation, age, sex, and obtaining ACA information from watching national television news were the most important predictors of attitudes about the ACA and its components. These results mirror national-level findings. Implications for implementing health care reform at the state-level are discussed.

## 1. Introduction

In March of 2010, President Barack Obama signed into law health care reform legislation focused on expanding access to affordable health care coverage, controlling rising health care costs, and improving health care delivery [1]. While few would argue against these goals, how best to achieve the goals and the merit of the current health care legislation has been intensely debated. A variety of opinion polls and statistics have fueled discussion over health care reform.

In June 2010, an Associated Press-GfK opinion poll found an overall increase in support for the Affordable Care Act (ACA). In particular, in one month's time, support increased 10% among men, 14% among adults between the ages 30 and 49, and 9% among Republicans, although the majority of Republicans (83%) still disapprove of the law [2]. Yet by October 2010, a Kaiser Family Foundation (KFF) report, summarizing eight national polls, found that people generally favored partial or full repeal of the law [3]. Public opinion in January of 2011 suggested the overall views regarding health care reform remained split with an even number of Americans supporting and opposing the law [4]. By November 2011, more Americans held an unfavorable view of the law than a favorable view [5]. Even after the June 2012 Supreme Court ruling in favor of the majority of the ACA provisions, US citizens polled in early July 2012 were evenly split between support for (41%) and against (41%) the ACA [6]. Throughout the 2012 presidential campaign, this trend remained consistent with 43% in favor and 39% opposed to reform. While health care reform was a factor in the race, it was not a driving factor and opinions did not vacillate much from the trends that began in April of 2010 [7]. The KFF Poll conducted in the wake of the governmental shutdown, October 2013, demonstrated a slight dip in the favorable views with 38% in favor of the legislation and 44% opposed [8]. The latest poll conducted in January 2014 indicated more unfavorable views (50%) than favorable views (34%), but in total, the trend numbers illustrate that attitudes toward the ACA have remained relatively stable even during substantial political events [9].

Despite the split in opinion regarding the ACA, in general, public opinion surveys suggest that Americans do favor many elements of the law. For example, in a January 2011 poll, 59% of individuals supported the provision that penalizes medium- and large-sized companies that do not offer health insurance to employees [4]. A November 2011 poll identified other elements of health care reform supported by the general public including understandable benefit summaries, small business tax credits and subsidies to aid in the purchase of insurance, and coverage even when individuals have preexisting conditions [5]. Even though Americans support health care reform elements, there is a general lack of support for tax increases to cover the cost of the reforms and questions remain about the impact of reform on current employer-sponsored insurance programs [10, 11].

While health care reform has been mandated at the national level, the execution of that reform will be realized at the state level. In addition, legal action requiring Supreme Court review of the legislation was advanced by state-level opposition. An April 2010 report by The Commonwealth Fund explored health care opinion leaders' views on health care reform, implementation, and postreform priorities. According to opinion leaders, the states' capacity to implement reform and the enforcement of the individual mandate requiring Americans to have health insurance are among the top barriers to implementation [12]. Lending support for this claim is the KFF's finding that the least favorable element of the health care reform law is the individual mandate [5].

While the preponderance of attitude research on health care reform has focused on national polls, this study examines attitudes held by Indiana residents over a three-year period. Our goal was to understand the views of Americans at the state level and how those views may be reflective of, or in opposition to, the ACA and national-level public opinion polls regarding health care reform. In addition, it is important to understand the information sources residents rely on as they learn about and form opinions about health care reform. This is a valuable analysis because reform at the state level requires an understanding of the attitudes of a state's citizenry, possible changes in attitudes over time, and the factors that may predict or contribute to specific attitudes for, or against, health care reform and/or its elements. To this end, we explored the following research questions.

RQ1a: How do residents in states view health care reform?
RQ1b: How have residents' views about health care reform changed?
RQ1c: Are the attitudes held by residents in a state different from national views regarding health care reform?
RQ2a: What information sources do residents use to learn about health care reform?
RQ2b: How influential are the information sources?
RQ3: Do factors, such as sex, race, age, political affiliation, insurance coverage, health care usage, and specific information sources predict support for, or against, elements of health care reform?

## 2. Materials and Methods

### 2.1. Selection and Description of Participants

The IRB approved survey was conducted in conjunction with Princeton Research Associates International of Princeton, NJ. A random sample of adults in the state of Indiana over the age of 18 was recruited during the last two weeks in November 2010, 2011, and 2012 to participate in the telephone, both cell phone and landline, survey. The recruitment procedure was consistent across the three years. To recruit respondents, a combination of landline and cellular random digit dial samples was used to identify the telephone numbers of and to contact adult Indiana residents. This systematic respondent selection technique enabled us to obtain a sample that closely represented the population in terms of age and gender when combined with the cell phone sample. For all the samples, the interviewers confirmed the respondent was a current resident of Indiana before administering the survey.

In 2010, the survey included questions on Indiana residents' views of the ACA in general, specific elements of the ACA, and demographic information. In 2011, the survey included the same polling questions regarding the residents' views of the ACA from 2010 so relevant comparisons could be made. In addition, we expanded the 2011 survey to include questions regarding the use of health care services. In 2012, we repeated the previous years' survey questions and added a question about the mandate to require individuals to purchase health care insurance and two questions regarding the variety of sources individuals used to obtain information about health care reform along with how influential the source was in the formation of the individuals' opinion. The response formats used for the survey items were multipoint Likert-type responses (e.g., favorable to unfavorable), closed-format responses (e.g., check one or check all that apply), and open-ended items to obtain frequency data (e.g., in the past 12 months, how many times have you, yourself, gone to an emergency room for medical treatment?). The survey was pilot tested prior to data collection.

Power analysis was conducted* a priori* and based on an eligible population of approximately 5 million adults in Indiana, a sampling error of ±5% at the 99% confidence level, it was determined that a sample of 498 adults would be needed to make inferences to the total population of adults in the state of Indiana. Our final sample size for all three years of the study far exceeded the required number (*n* = 601 in 2010, *n* = 607 in 2011, and *n* = 602 in 2012).

### 2.2. Data Analysis

Data were analyzed using STATA 12. Using weighted data, we computed descriptive statistics to investigate the responses to the survey items as well as the demographic and background characteristics of the respondents. We computed chi-square tests and conducted binary logistic regression analyses using the weighted data to assess the association between the dependent variables (attitudes about health care reform, health services usage, and information sources) and independent predictor variables (race, gender, age, insurance status, education, and political affiliation of the respondents). A two-stage weighting procedure was used. The first stage of weighting corrected for different probabilities of selection associated with the number of adults in each household and each respondent's telephone usage patterns. The second stage of weighting balanced sample demographics to population parameters. The sample was balanced to match parameters for sex, age, education, race, Hispanic origin, region, population density, number of adults in the household, and telephone usage.

## 3. Results

### 3.1. Demographic and Background Characteristics of Study Participants

Table 1 presents the descriptive frequencies for the respondent characteristics across all three years of the study. In general, the samples were similar from year to year; the respondents represented in the samples were predominately white, non-Hispanic, covered by some form of health insurance, and evenly split between men and women. The weighted average age of the respondents was approximately 47 years (2010: $\overset{-}{x}=47.56$ years, std error = ±0.84; 2011: $\overset{-}{x}=47.12$ years, std error = ±0.80; and 2012: $\overset{-}{x}=47.77$ years, std error = ±0.84). The most frequently reported household income range was $30,000 to $50,000. Most of the respondents held a high school degree and approximately a quarter of the respondents had completed some college. Political affiliation was generally consistent across the three years of the study.

**Figure 1:**
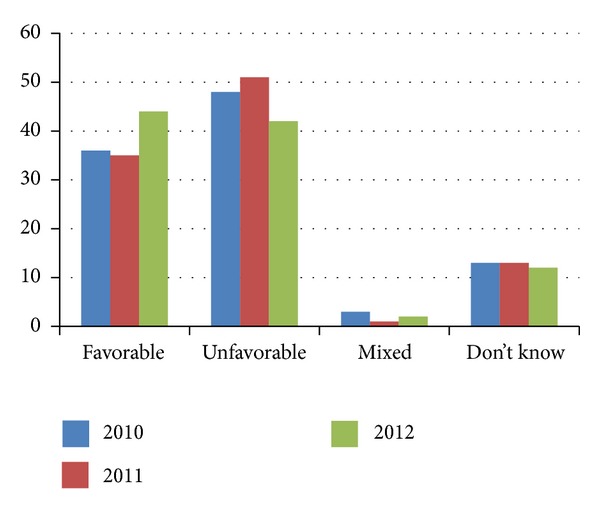
Indiana residents' views of the Affordable Care Act 2010–2012.

In this study, we asked respondents whether they supported the ACA or not. The resulting data demonstrated a change in Indiana residents' views of the ACA (see Figure 1). In 2010, 48% of Indiana residents expressed an unfavorable view of the ACA compared with 51% in 2011, and 42% in 2012. The initial increase in negative sentiment may have been the result of a decrease in the number of Indiana residents who reported having a mixed view of the ACA in 2010, while the subsequent increase in positive sentiment in 2012 was a shift from unfavorable to favorable views. The percentage of those reporting a “mixed view” of the ACA fell from 3% to 1% from 2010 to 2011 and increased to 2% in 2012. There was not a statistically significant difference in the “mixed view” category among the years. The Kruskal-Wallis equality of populations rank test indicated a statistically significant difference at the 1% level among the years regarding overall changes in views of the ACA (*χ* ^2^ = 13.694, *P* = 0.001). Follow-up chi-square analyses indicated that the increase in the proportion of individuals reporting favorable views over the three years was the greatest contributing factor to the overall significant chi-square result (favorable proportional changes *χ* ^2^ = 9.315, *P* = 0.001; unfavorable proportional changes *χ* ^2^ = 4.797, *P* = 0.091).

In regard to health care use and perceptions of the ACA during 2011 and 2012, there was a difference in overall support. Generally, respondents who had used some form of health care service at least once during each year of the survey reported more support for the ACA (2011: 35%; 2012: 45%) than those respondents who did not seek or require health services (2011: 29%; 2012: 38%), *χ* ^2^ = 4.309, *P* = 0.038.

In our study, in addition to indicating their overall view of the ACA, respondents were asked about key elements of the ACA, specifically if making coverage affordable, ensuring coverage for everyone, mandating that preexisting conditions be accepted by insurance companies, providing coverage for children until age 26, and the individual mandate requiring people to purchase health insurance were important. In 2010, despite 48% reporting an unfavorable view of the ACA, 96% of Indiana residents revealed that making coverage affordable was important. Similarly, 80% and 91% of Indiana residents in 2010 felt that ensuring coverage for everyone and the coverage for preexisting condition mandates were important. When asked about the importance of providing coverage for children until age 26, 69% of Indiana residents felt that was important. In 2011 and 2012, despite 51% and 42% reporting an unfavorable view of the ACA, respectively, 95% and 93% of Indiana residents felt affordable health care coverage was important, 83% and 81% supported ensuring coverage for everyone, 90% of residents in both 2011 and 2012 felt insurance companies should cover preexisting conditions, 70% and 77% felt children should be covered until age 26, and in 2012, 63% of Indiana residents supported the individual mandate requiring people to purchase health insurance. While the majority of changes in the percentage of Indiana residents reporting that these elements were important from 2010 to 2012 are small and not statistically significant, there is a significant difference among the years in the percentage reporting they felt that children should be covered until the age of 26 at the 1% level (*χ* ^2^ = 13.896, *P* = 0.001). Overall, from 2010 to 2012, despite a general negative attitude toward the ACA, the majority of Indiana residents consistently support key elements of the legislation.

While making insurance coverage affordable was viewed as important by more than 9 out of 10 Indiana residents from 2010 to 2012, the importance of affordable coverage, or any of the ACA elements, may be viewed differently based on the respondent's health insurance status. Therefore, we stratified the respondents by insurance status to determine if there were attitude differences. In 2010, 36% of insured respondents had a favorable view of the ACA compared to 32% of uninsured respondents. In 2011, the respective comparisons were 34% of insured respondents versus 36% of uninsured respondents and in 2012, 44% versus 42%.

In general, providing insurance coverage for everyone was important to approximately 8 in 10 Indiana residents. Between 2010 and 2012 support for this element of the ACA initially increased from 80% to 83% and then decreased to 81%. In 2010, support for this provision was higher among uninsured Indiana residents (87%) compared to insured Indiana residents (78%), and this difference was statistically significant, *χ* ^2^ = 4.557, *P* = 0.031. In 2011, while the percentage of uninsured respondents who felt ensuring coverage for everyone remained constant (87%), the number of insured respondents reporting that providing coverage for everyone was important increased to 82%. By 2012, nearly equal percentages of insured and uninsured respondents felt providing coverage for everyone was important (81% and 80%, resp.).

The ACA elements receiving the least amount of support from Indiana residents were allowing children up to age 26 to be covered by their parents' health insurance whether or not they were in college and the individual mandate. In 2010 and 2011, approximately 69% of respondents supported insuring children up to age 26. In 2012, the percentage of respondents indicating support for this element of the ACA rose to 77%. This increase in 2012 was statistically significant, *χ* ^2^ = 13.896, *P* = 0.001. Across the three years, support for covering children up to age 26 was relatively consistent with approximately 71% of insured respondents and 75% of uninsured respondents in favor of this element. In 2012, 63% of respondents felt the individual mandate was important. Analysis of attitude difference by insurance status revealed 64% of insured respondents felt the individual mandate was important compared to 59% of uninsured respondents.

In an effort to clarify our understanding of the public's opinion of the ACA, the 2011 and 2012 surveys were expanded to include questions that would elicit information regarding additional confounders. Therefore, these surveys included questions regarding health care utilization. Health care utilization questions asked respondents how many times during the past 12 months they had visited a doctor, stayed overnight in the hospital, visited the hospital for outpatient care, or went to the emergency room. The most interesting results revolved around emergency room use and physician visits.

**Figure 2:**
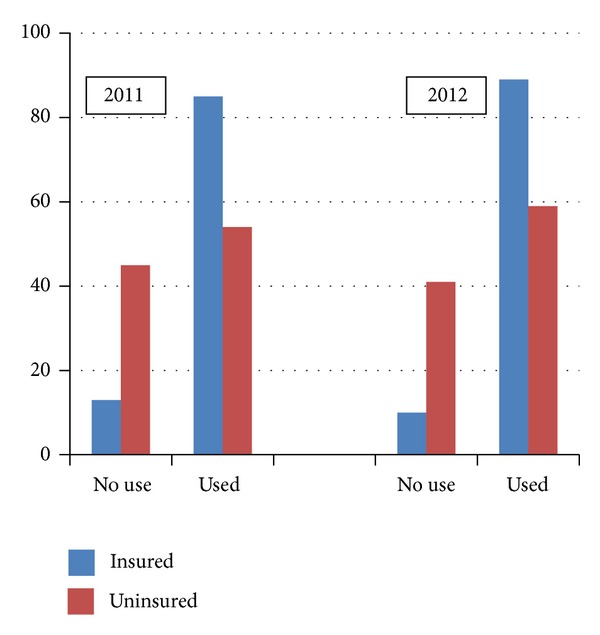
Physician visits by insurance coverage 2011-2012. Note: the data reported in Figure 2 is based on only those respondents who answered both survey questions—physician use and insurance status.

In 2011 and 2012, equivalent percentages of insured and uninsured respondents reported utilizing the ER at least once during the past 12 months (2011: 29% versus 30%; 2012: 27% versus 29%). Since ER use is not necessarily planned and, in general, is a more expensive point-of-access into the health care system, we polled Indiana residents on their use of physician visits, a less expensive health care service (Figure 2). Insured Indiana residents were more likely to use physician services compared to uninsured Indiana residents. Of the Indiana residents polled in 2011 and 2012, 85% and 89% of insured Indiana residents reported visiting a physician in the last 12 months compared to 54% and 59% of Indiana residents who were not insured. These differences were significant, *χ* ^2^ = 45.82, *P* < 0.001 and *χ* ^2^ = 44.21, *P* < 0.001.

### 3.2. Logistic Analysis of Predictors

For the data from 2010 to 2012, we explored how the respondent characteristics predicted attitudes about the ACA using logistic regression modeling to control for confounders that could influence the views of individuals (Table 2). Each categorical independent variable was recoded to create a dichotomous variable (e.g., race was recoded into white versus nonwhite). The models presented in Table 2 report the adjusted odds ratios which controlled for potential confounders. All models were significant at *P* < 0.01 and *F*-values ranged from 2.73 to 15.00. We found that females (AOR = 1.48, *P* < 0.01), nonwhites (AOR = 1.72, *P* = 0.02), Democrats (AOR = 2.75, *P* < 0.01), and insured individuals (AOR = 1.60, *P* = 0.04) were more likely to have a general favorable attitude towards health care reform. Older Indiana residents (AOR = 0.99, *P* = 0.04) and Republicans (AOR = 0.41, *P* < 0.01) were less likely to have a favorable attitude towards health care reform.

When asked about the importance of health care affordability, females (AOR = 1.90, *P* = 0.04) and Democrats (AOR = 31.33, *P* < 0.01) were more likely to affirm that attitude, while the opposite was true as income (AOR = 0.99, *P* < 0.01) increased. When asked about the importance of preexisting condition coverage, females (AOR = 1.99, *P* = 0.01) were more likely to support that view. Females (AOR = 2.25, *P* < 0.01), nonwhites (AOR = 4.31, *P* = 0.02), and Democrats (AOR = 6.38, *P* < 0.01) were more likely to view providing health care coverage for everyone as important, while Republicans (AOR = 0.47, *P* < 0.01) were less likely to view this as important and support for this element decreased as age (AOR = 0.98, *P* < 0.01) and income (AOR = 0.99, *P* = 0.02) increased. Females (AOR = 2.01, *P* < 0.01) and Democrats (AOR = 2.29, *P* < 0.01) were more likely to support coverage of children up to age 26 and, in general, support for this element increased from 2010 to 2012 (AOR = 1.28, *P* < 0.01). Consistent with previous findings, support for insuring children up to age 26 decreased as age (AOR = 0.97, *P* < 0.01) increased. For the individual mandate requiring people to purchase health insurance introduced in 2012, females (AOR = 1.97, *P* < 0.01), nonwhites (AOR = 7.83, *P* < 0.01), and Democrats (AOR = 2.77, *P* < 0.01) were more likely to support this element.

For the 2011 and 2012 surveys, we explored whether the independent respondent characteristic variables predicted health services usage (Table 3). As in Table 2, we present the adjusted analysis controlling for potential confounders. All models were significant with *F*-values ranging from 2.21 to 9.77 and *P* values ranging from .019 to <.001. The results indicate visiting a physician in the past 12 months was strongly associated with females (AOR = 1.84, *P* < 0.01), older age (AOR = 1.01, *P* = 0.01), Democrats (AOR = 1.67, *P* = 0.05), and having health insurance coverage (AOR = 5.32, *P* < 0.01). Those with health insurance were more likely to have stayed overnight in the hospital (AOR = 3.46, *P* < 0.01) or to have been treated as an outpatient in the hospital (AOR = 2.62, *P* < 0.01). The likelihood of an overnight stay increased as age increased (AOR = 1.01, *P* = 0.03), yet the likelihood of an ER visit decreased as age increased (AOR = 0.99, *P* = 0.03). Finally, the likelihood of overnight hospital stays and ER visits decreased as income increased (AOR = 0.99, *P* < 0.001).

These data provide intriguing insights into individuals' views about the ACA and what participant characteristics are associated with these views. The 2010 and 2011 data, however, does not provide insight into what information sources may be contributing to the creation of these views. Thus, in 2012, we added survey questions to ascertain what communication sources individuals used to obtain information about the ACA and whether the information they obtained from those sources contributed to their opinions regarding the ACA and its elements.  Table 4 lists the information sources, the percentage (weighted data) of individuals who obtained information from those sources and the percentage (weighted data) of individuals who identified this source as important to their opinion formation.

As demonstrated in Table 4, individuals in Indiana, and we would argue across the nation, received messages regarding the ACA from a variety of sources; the top four sources that respondents reported contributing most to their opinion formation were national news programs, websites, family members, and their own reading of the legislation. To determine if these four sources were predictive of overall ACA views and support for the individual elements, we employed logistic regression modeling (Table 5) using only the 2012 data. Each model was significant with *F*-values ranging from 2.11 to 5.38 and *P* values ranging from 0.017 to <0.001.

Review of the logistic regression results indicated that individuals who relied on national television news as an ACA information source generally held more favorable attitudes toward health care reform and its provisions (AOR ranging from 1.70 to 4.11 and *P* values ranging from <0.001 to 0.040) and individuals who obtained information from the Internet held more favorable views toward affordable coverage for all (AOR = 2.77, *P* = 0.050). Surprisingly family members, while identified as a significant information source, only influenced individuals' views regarding the preexisting condition provision. Generally, those individuals who relied on family members' opinions held less favorable views regarding coverage for preexisting conditions (AOR = 0.38, *P* = 0.044). While over a third of the respondents reported having read the legislation and that the information they gained was very or somewhat important to their opinion formation, this information source was not significant in the model.

## 4. Discussion

Health care reform is a significant policy issue facing the United States. The issue remains politically charged as evidenced by our findings and national level studies. State-level distinctions are essential for state government officials to understand as they make ACA implementation decisions and choices [13]. State-level support is necessary for success since many of the individual regulations found in the ACA will be implemented by the states. Our study shows that, in Indiana, there is overwhelming support for three of the five individual provisions of the ACA addressed in this research and strong support for the remaining two provisions. Individuals in our sample have formed their perceptions of the overall ACA based primarily on political affiliation and relied heavily on information gained from the national news media. The partisan nature of this issue and lack of support from the states are further evidenced by the arguments brought before the Supreme Court of the United States by some states including Indiana.

Amidst the political debate, residents of Indiana strongly support the main provisions of the ACA. This could serve as a foundation to grow further bipartisan and grass roots support for health care reform. Yet, as demonstrated in the results, people's attitudes toward reform ideals and the ACA are inconsistent. The cause of this inconsistency is beyond the scope of the current findings and is an area in need of future research; however, our 2012 investigation into the influence of varying information sources offers a necessary first step when determining factors contributing to these inconsistencies.

Based on our 2010 and 2011 results, we believed that those individuals who reported actually reading the legislation were more likely to form an opinion, good or bad, based on the merits of the legislation. In contrast, reliance on secondary sources such as the media, politicians, or members of social networks for information regarding any legislative action could lead to the lack of support for legislation that in fact is consistent with an individual's views. The results of the 2012 analysis indicate that one's own reading of the legislation is not associated with opinion formation and information individuals obtain from the national news media is associated with positive attitude formation. This finding may be a reflection of the complexity inherent in the formal articulation of the health care reform legislation and could be argued of legislative documents in general. Individuals must be able to comprehend and process information to make informed decisions and opinions regarding the issue at hand. The national news media is able to provide a necessary first-interpretation of the legislation that then enables individuals to process the ideas and formulate opinions. While more research is needed into the cause of these inconsistencies, it is clear that the national news media continues to be a powerful influence in public opinion formation [14–16].

The results of our study should be viewed in light of several potential limitations that are generic for our study design and specific for our study content. First, the information sought in this study had to be obtained through self-reported perceptions and behaviors. Some individuals may not have remembered events correctly or may have provided socially desirable responses to some items, both being potential threats to the internal validity of the findings. Second, the monothematic nature of the questionnaire may have caused some individuals to think about the topic in a unique manner; if so, this would be a threat to the internal validity of the findings. Third, this study used a cross-sectional design. Thus, no cause and effect conclusions can be drawn from these results. Fourth, even though we had a reasonable sample size and included the questions of key relevance, it is still possible that a larger sample size and other unmeasured variables could better explain support for and perceptions of the ACA.

## 5. Conclusions and Policy Implications

Nationwide, and in Indiana specifically, this inconsistency in attitudes toward the ACA and attitudes about health care ideals must be addressed if health care reform and subsequent policies are to be successful. In the wake of the Supreme Court decision upholding much of the ACA, states will have to move forward with an implementation plan. One element of the plan must include clearly communicating to the citizenry how specific elements of the ACA align with their current views. Reform acceptance will likely require additional education about health care reform benefits and limitations. Previous research conducted by Nixon and Aruguete established a link between knowledge of the health care delivery system and negative attitudes toward the health care delivery system. In the Nixon and Aruguete study, these attitudes predicted support for health care reform [17]. Perhaps for reform to succeed there needs to be education about the reform elements along with education about current health care delivery system failures.

Clearly, the acts of passing the legislation by Congress and the upholding of the legislation by the Supreme Court cannot guarantee the acceptance of the ACA. There are many challenges associated with implementation of the ACA. A goal of the ACA is to increase Americans' access to health care services. The results of our study demonstrate that residents with health insurance are more likely to visit the doctor, stay overnight in the hospital, and seek outpatient hospital care. Questions remain regarding whether the current health care system can meet the increase in demand for care and services that will occur when the ACA is fully implemented. While more physicians are projected to enter the workforce in the upcoming years, the rate of growth is not expected to meet the 22% increase in demand projected for 2020 [18] and experts have identified the inadequate supply of primary care physicians as a main barrier to ACA implementation [12].

Along with addressing health care delivery barriers, policy makers will have to attend to the negative overall view held by the citizenry. It is important to understand, predict, and address the negative attitudes to promote effective implementation of any new legislation. Failure to do so will result in energies focused on fighting these policies versus determining which policy processes effectively and efficiently meet the health care needs of the public.

## Figures and Tables

**Table 1:** Respondent characteristics by year: weighted frequencies.

Characteristic	Categories	2010	2011	2012
Gender	Male	49%	48%	49%
	Female	51%	52%	51%

Race	White	88%	87%	88%
	Nonwhite	11%	12%	10%

Ethnicity	Hispanic	1%	3%	3%
	Non-Hispanic	98%	96%	96%

Health insurance	Yes	82%	81%	85%
	No	17%	18%	14%

Income	<$20 K	16%	21%	18%
	$20 K–$30 K	13%	16%	16%
	$30 K–$50 K	22%	20%	19%
	$50 K–$75 K	16%	12%	18%
	$75 K–$100 K	10%	8%	11%
	>$100 K	8%	11%	11%

Education	Some high school	12%	11%	10%
	High school degree	41%	42%	38%
	Some college	26%	26%	29%
	College degree	13%	12%	14%
	Graduate degree	9%	9%	9%

Political affiliation	Republican	29%	32%	26%
	Democrat	28%	27%	30%
	Independent	33%	33%	36%

Note: categorical percentages may not sum to 100%, given that some respondents selected not to answer some of the demographic questions.

**Table 2:** Predictors of attitudes towards health care reform and five provisions.

	Overall impression of health care reform^+^		Affordable coverage		Coverage for everyone		Preexisting conditions		Coverage of children up to age 26		Individual mandate requiring health insurance	
Predictors	AOR∗	*P *	AOR	*P *	AOR	*P *	AOR	*P *	AOR	*P *	AOR	*P *
Female	**1.48**	**0.005**	**1.90**	**0.035**	**2.25**	**<0.001**	**1.99**	**0.01**	**2.01**	**<0.001**	**1.97**	**0.004**
Nonwhite	**1.72**	**0.024**	0.61	0.436	**4.31**	**0.017**	0.54	0.14	1.45	0.185	**7.83**	**0.002**
Age	**0.99**	**0.039**	0.99	0.162	**0.98**	**0.001**	1.00	0.30	**0.97**	**<0.001**	0.99	0.196
Income	1.00	0.870	**0.99**	**0.004**	**1.00**	**0.020**	1.00	0.79	1.00	0.222	0.99	0.422
Some college	0.81	0.167	0.78	0.425	0.86	0.408	1.07	0.79	0.80	0.132	0.93	0.782
Democrat	**2.75**	**<0.001**	**31.3**	**<0.001**	**6.38**	**<0.001**	1.89	0.10	**2.29**	**<0.001**	**2.77**	**0.001**
Republican	**0.41**	**<0.001**	0.85	0.619	**0.47**	**<0.001**	0.78	0.36	0.83	0.198	0.80	0.375
Insured	**1.60**	**0.036**	1.55	0.294	0.94	0.802	1.45	0.27	0.99	0.958	1.57	0.187
Year	1.17	0.068	0.71	0.074	1.02	0.868	0.91	0.53	**1.28**	**0.002**	NA^†^	NA

^+^The specific questions asked to elicit attitude data are as follows: What is your overall impression of the health care law passed by Congress? Is it…?, How important is making health care more affordable?, How important is ensuring health coverage for everyone?, How important is prohibiting insurance companies from cancelling health plans due to preexisting conditions?, How important is allowing children up to age 26 to be covered by their parents' health insurance whether or not they are in college?

∗AOR: adjusted odds ratio implies controlling for age, gender, race, education, insurance status, and political affiliation. These are for association of favorable attitudes with independent predictors. The binary dependent variable was “attitude”-favorable (1) or unfavorable (0) or important (1) or unimportant (0). *P* values for odds (2 df, Wald's *χ* ^2^ test).

^†^Data regarding respondents' attitudes toward the individual mandate to purchase health insurance was only collected in 2012.

**Table 3:** Predictors of health care usage.

	Doctor's visit^+^		Overnight hospital stay		Outpatient hospital care		ER visit	
Predictors	AOR∗	*P *	AOR	*P *	AOR	*P *	AOR	*P *
Female	**1.84**	**0.004**	1.09	0.689	1.12	0.440	1.08	0.640
Nonwhite	1.19	0.626	0.66	0.251	0.87	0.590	1.27	0.372
Age	**1.01**	**0.013**	**1.01**	**0.034**	1.00	0.621	**0.99**	**0.029**
Income	1.00	0.380	**0.99**	**>0.001**	1.00	0.441	**0.99**	**>0.001**
Some college	0.97	0.913	0.99	0.953	1.04	0.813	0.98	0.925
Democrat	**1.67**	**0.049**	1.55	0.057	1.19	0.349	1.00	0.998
Republican	1.01	0.974	1.00	0.993	0.84	0.360	0.71	0.088
Health insurance	**5.32**	**>0.001**	**3.46**	**0.001**	**2.62**	**>0.001**	1.48	0.088
Year	1.31	0.193	0.96	0.835	0.97	0.854	0.90	0.502

^+^The specific questions asked to elicit health services usage data are as follows: In the past 12 months, how many times have you, yourself, made a doctor visit?, In the past 12 months, how many times have you, yourself, had an overnight stay in a hospital?, In the past 12 months, how many times have you, yourself, gone to the hospital for outpatient care, not including ER visits?, In the past 12 months, how many times have you, yourself, gone to an emergency room for medical treatment? Our usage variables take two values with 0 indicating zero usage and 1 indicating at least one usage.

∗AOR: adjusted odds ratio implies controlling for age, gender, race, education, insurance status, and political affiliation. These are for association of favorable attitudes with independent predictors. The binary dependent variable was “usage of health services” with 0 indicating no health care use.

**Table 4:** Information sources and importance 2012.

Information source	% of respondents obtaining ACA information from the source	% of respondents reporting the information was important to opinion formation	
		Very	Somewhat
Local daily newspaper	38%	12%	47%
National newspaper	28%	17%	41%
Local TV news	53%	19%	46%
National TV news	68%	25%	46%
Radio	32%	23%	42%
Websites	39%	27%	48%
Family nembers	50%	34%	36%
Friends	45%	14%	49%
Coworkers	31%	15%	40%
Politicians	32%	15%	48%
Religious leaders	13%	21%	42%
Personal reading of legislation	36%	46%	36%

Note: respondents were able to identify multiple information sources.

**Table 5:** Information source as predictors of attitudes towards health care reform.

	Overall impression of health care reform		Affordable coverage		Coverage for everyone		Preexisting conditions		Coverage of children up to age 26		Individual mandate requiring health insurance	
Predictors	AOR	*P *	AOR	*P *	AOR	*P *	AOR	*P *	AOR	*P *	AOR	*P *
Female	**2.05**	**0.007**	1.82	0.230	**2.24**	**0.012**	1.82	0.149	1.11	0.679	**2.10**	**0.002**
Nonwhite	2.59	0.083	+				0.51	0.382	0.99	0.983	**8.78**	**0.001**
Age	1.00	0.744	1.00	0.973	0.99	0.286	0.99	0.566	**0.98**	**0.002**	0.99	0.296
Income	1.00	0.136	1.00	0.276	1.00	0.537	1.00	0.904	**0.99**	**0.001**	1.00	0.451
Some college	0.94	0.832	0.75	0.566	1.28	0.460	0.86	0.726	**0.48**	**0.005**	0.97	0.905
Democrat	**5.98**	**0.000**			**4.80**	**0.014**	2.03	0.340	**3.09**	**0.003**	**2.62**	**0.002**
Republican	**0.41**	**0.005**	0.46	0.146	**0.54**	**0.044**	0.47	0.087	0.74	0.285	0.78	0.337
Insured	1.06	0.903	1.42	0.574	1.35	0.515	**3.26**	**0.047**	0.98	0.955	1.51	0.247
National TV news	**1.96**	**0.029**	**4.11**	**0.005**	**3.24**	**<0.001**	**2.47**	**0.040**	**2.11**	**0.006**	**1.70**	**0.034**
Websites	0.82	0.433	**2.77**	**0.050**	0.65	0.174	0.49	0.132	0.67	0.162	1.18	0.507
Family members	0.82	0.460	0.47	0.143	0.65	0.147	**0.38**	**0.044**	0.81	0.432	0.81	0.386
Personal reading of legislation	1.09	0.735	0.58	0.198	0.59	0.085	1.59	0.308	1.48	0.149	0.66	0.098

^+^Blank cells indicate that in the model calculation the variable did not predict the attitude and was ultimately omitted.
